# Absence of flexor carpi radialis identified during volar approach for fixation of distal radius fracture: a case report

**DOI:** 10.1186/s13104-018-3348-z

**Published:** 2018-04-11

**Authors:** Tomoyo Irie, Makoto Motomiya, Norimasa Iwasaki

**Affiliations:** 1 Department of Orthopaedic Surgery, Faculty of Medicine and Graduate School of Medicine, Hokkaido University, Sapporo, 060-8638 Japan; 2 Department of Orthopaedic Surgery, Obihiro Kosei Hospital, Nishi 6 Minami 8-1, Obihiro, 080-0016 Japan

**Keywords:** Flexor carpi radialis, Anomaly, Distal radius fracture, Volar approach, Flexor carpi radialis brevis

## Abstract

**Background:**

Volar locking plate fixation of distal radius fractures is commonly performed because of its good clinical outcomes. The flexor carpi radialis (FCR) approach is one of the most popular approaches to dissecting the volar side of the distal radius because of its simplicity and safety. We describe an extremely rare case of an absent FCR identified during a volar approach for fixation of a distal radius fracture.

**Case presentation:**

A 59-year-old woman with distal radius fracture underwent surgery using the usual FCR approach and volar locking plate. We could not identify the absence of the FCR tendon preoperatively because of severe swelling of the distal forearm. At first, we wrongly identified the palmaris longus tendon as the FCR because it was the tendinous structure at the most radial location of the volar distal forearm. When we found the median nerve just radial to the palmaris longus tendon, we were then able to identify the anatomical abnormality in this case. To avoid iatrogenic neurovascular injuries, we changed the approach to the classic Henry’s approach.

**Conclusions:**

Although the FCR approach is commonly used for fixation of distal radius fractures because of its simplicity and safety, this is the first report of complete absence of the FCR during the commonly performed volar approach for fixation of a distal radius fracture, to our knowledge. Because the FCR is designated as a favorable landmark because of its superficially palpable location, strong and thick structure, and rare anatomical variations, there is the possibility of iatrogenic complications in cases of the absence of the FCR. We suggest that surgeons should have a detailed knowledge of the range of possible anomalies to complete the fixation of a distal radius fracture safely.

## Background

Volar locking plate fixation of distal radius fractures is commonly performed because of its good clinical outcomes [1, 2]. The flexor carpi radialis (FCR) approach is one of the most popular approaches to fixing distal radius fractures from the volar side because of its simplicity and safety [2, 3]. However, surgeons have reported various types of anatomical variation and anomalies of the distal volar forearm structures including, among others, bifurcation of the median nerve, an anomalous course of the palmar cutaneous branch (PCB) of the median nerve, the accessory muscles of the flexor tendon, and have warned about consequent iatrogenic injuries during surgery [4, 5].

Here, we describe an extremely rare case of the absence of the FCR, identified during the volar approach for fixation of a distal radius fracture.

## Case presentation

**Fig. 1:**
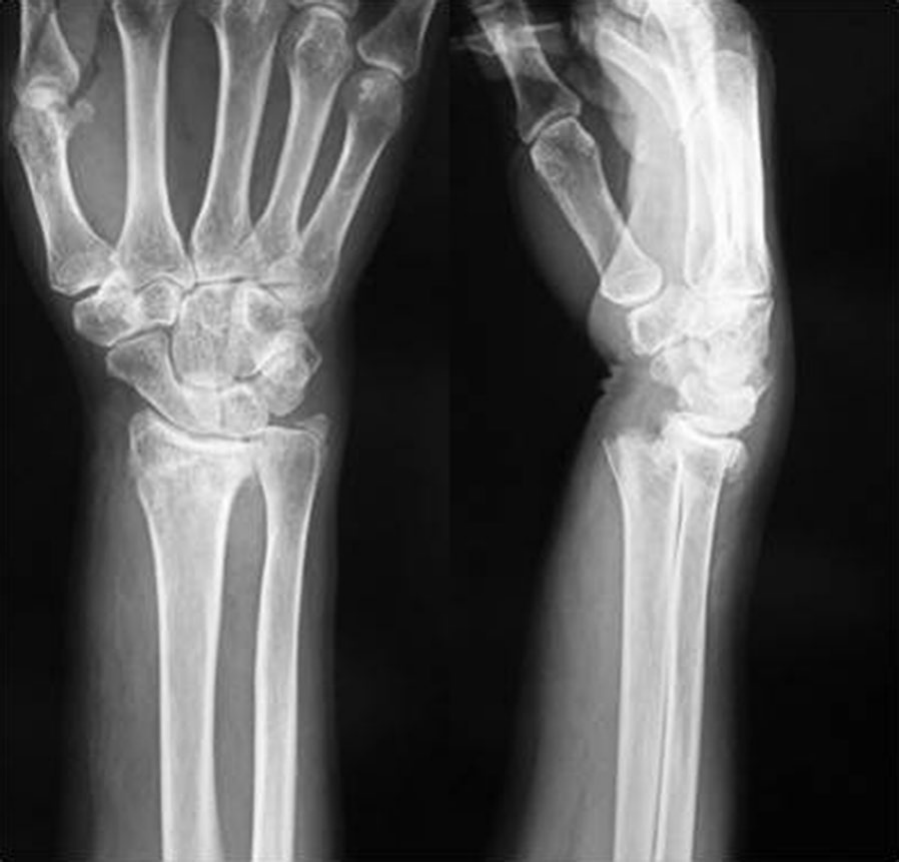
X-rays of the right hand at the first examination showing the distal radius fracture with a rim fragment

**Fig. 2:**
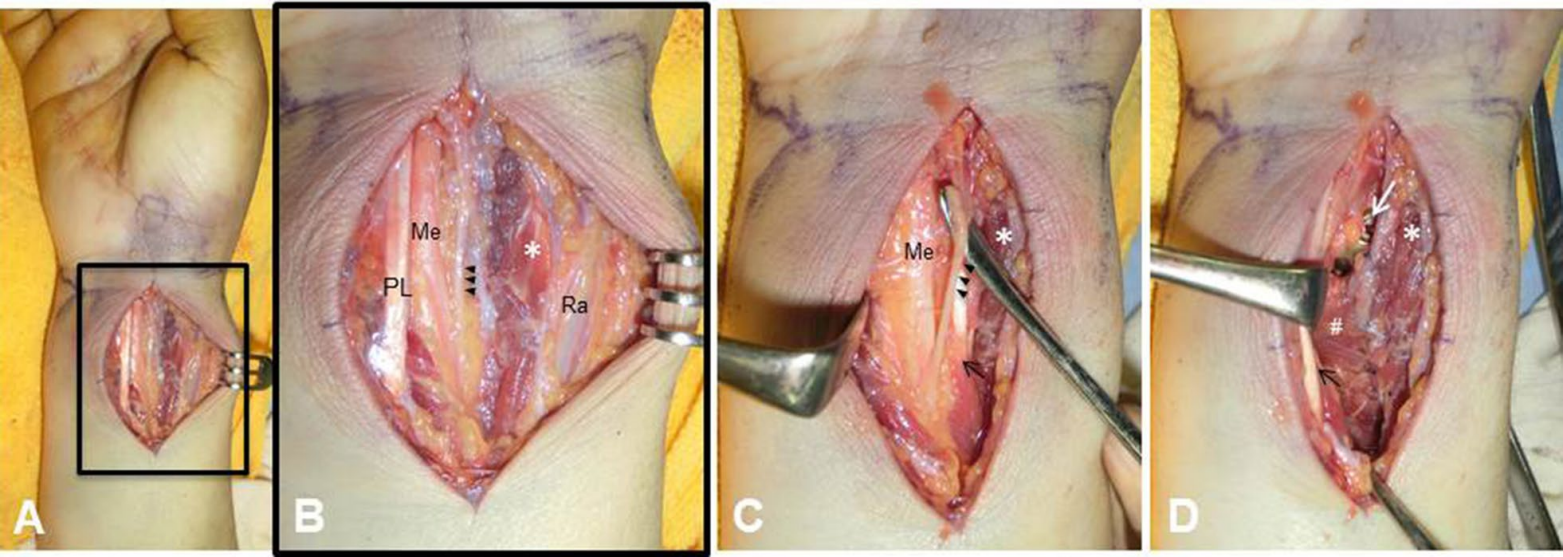
**A**, **B** The FCR tendon could not be identified using the commonly performed volar approach. **C** The intraoperative findings after retracting the palmaris longus tendon (PL). The median nerve (Me) and its palmar cutaneous branch (arrowhead) were difficult to identify without the FCR as a landmark. **D** The FCRB (asterisk) was identified just radial to the pronator quadratus muscle (hash mark) after retracting the flexor pollicis longus tendon (black arrow). (Ra: radial artery, white arrow: locking plate)

A 59-year-old right-handed female desk worker with no significant past medical history suffered a distal radius fracture (AO classification 23-A2 type) of the right hand (Fig. 1), fracture of the right clavicle, and multiple rib fractures with hemopneumothorax as the result of a high-energy traffic accident. The patient underwent initial treatment for her life-threatening conditions, including thoracic drainage and distal radius fracture fixation with plaster. Five days after the injury, the patient’s condition was no longer critical, and she underwent the definitive treatment of open reduction and internal fixation of the fractures of her clavicle and distal radius under general anesthesia. We had planned to fix the distal radius fracture using the usual FCR approach and a special plate designed for a volar rim fragment of the distal radius. We could not identify the absence of the FCR tendon preoperatively because of severe swelling of the distal forearm. At first, we wrongly identified the palmaris longus (PL) tendon as the FCR because it was the tendinous structure at the most radial location of the volar distal forearm. When we found the median nerve just radial to the PL tendon, we were then able to identify the anatomical abnormality described in this case (Fig. 2A, B). Therefore, we changed the approach to the classic Henry’s approach after we had identified and gently protected the PCB. When we exposed and protected the radial artery through the same incision, we noticed another abnormality when dissecting the deep layers of the volar distal forearm (Fig. 2C): after retracting the flexor pollicis longus tendon to the ulnar side, we found that an abnormal muscle existed just radial to the pronator quadratus (PQ) muscle (Fig. 2D). We diagnosed the abnormal tendon as the flexor carpi radialis brevis (FCRB) because of the wrist flexion and slightly radial deviation observed with the traction of the tendon, as described in a previous report [5]. We exposed the radius between the PQ and the FCRB muscles and fixed the fracture rigidly using a rim-fragment locking plate (2.4 mm variable angle LCP Rim Distal Radius Plate; Depuy Synthes Co., Tokyo, Japan).

**Fig. 3:**
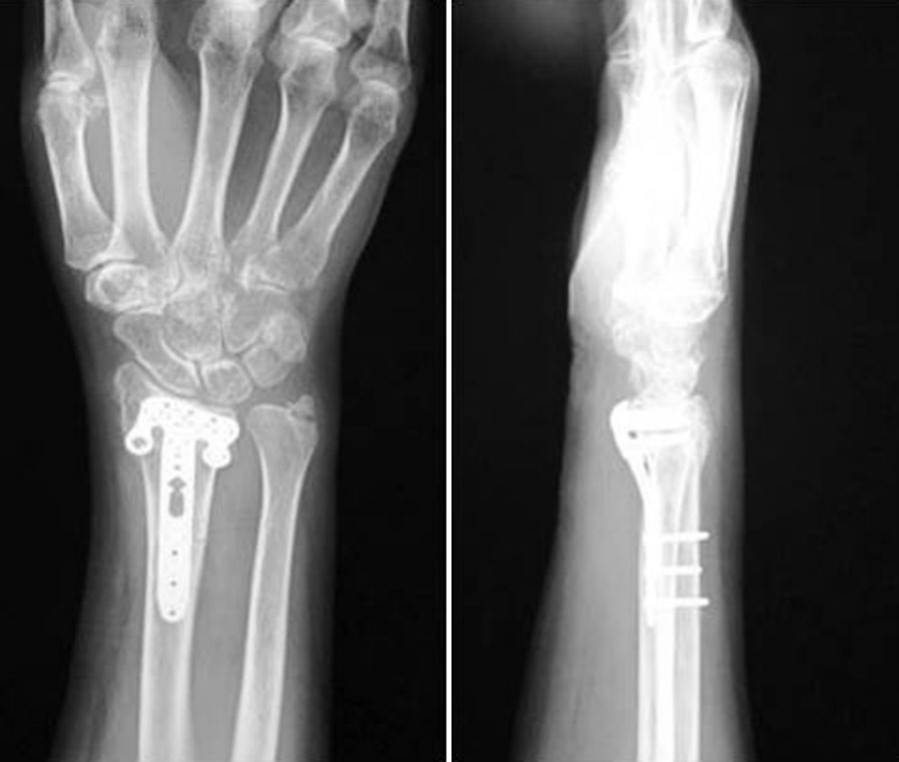
X-rays 4 months after surgery showing good reduction and stabilization with the locking plate for the rim fragment

Four months after the operation, the patient had no pain or neurologic problems and the X-rays showed complete bone union of the distal radius fracture (Fig. 3). Although a slight extension and flexion contracture of the wrist joint remained, the patient returned to her desk work without any disability. We checked for the existence of an FCR tendon in the opposite forearm, and could identify the thick FCR tendon by palpation and ultrasonic examination.

## Discussion and conclusions

The FCR originates from the medial epicondyle of the humerus and inserts into the trapezius, the second metacarpal, and the third metacarpal bones, and functionally contributes to the motion in flexion and the radial deviation of the wrist joint [6]. Although there have been reports of anatomical variations in its origin, insertion, and the presence of additional slips, complete absence of the FCR is an extremely rare anomaly that has been reported only twice in English-language reports [7, 8]. Rumball et al. [7] reported the absence of the FCR in a young boy undergoing tendon transfer for the reconstruction of postero interosseous nerve palsy, and Sofos et al. [8] reported the anomaly in a case of ligament reconstruction and tendon interposition arthroplasty for thumb carpometacarpal joint arthritis. Both reports focused on the need to avoid using a donor tendon in cases of absent FCR. However, the FCR also plays a very important role as a landmark in the distal forearm, and most surgery on the distal volar forearm is likely to be performed based on the location of the FCR [3, 9]. To our knowledge, this is the first report of an absent FCR identified during the commonly performed volar approach for fixation of a distal radius fracture.

Because important neurovascular structures exist close together in the distal volar forearm, there have been many complications reported for volar plating of distal radius fractures, including injuries to the median nerve, the PCB of the median nerve, and the radial artery [4, 10]. The FCR is designated as a favorable landmark because of its superficially palpable location, strong and thick structure, and rare anatomical variations. To avoid iatrogenic neurovascular injuries, detailed anatomical studies of the distal volar forearm have been based on the FCR [3, 9]. However, in rare cases where the FCR is absent, there is the possibility of misidentifying the PL as the FCR based on its most radial location in the distal volar forearm, especially under conditions of soft-tissue swelling around the distal forearm resulting from high-energy force. We suggest that the surgical approach should be changed to the classic Henry’s approach when the surgeon observes serious anatomical anomalies in the distal volar forearm [4].

In the present case, in addition to the absence of the FCR, we also identified an anomalous FCRB muscle. In general, the FCRB is considered to be an accessory muscle of the FCR that arises from the volar surface of the radius and inserts at various sites, including the base of the metacarpal bone, trapezium, and capitate [11]. Although this muscle functions to allow weak flexion of the wrist, it seems to be less important in normal function [5]. Although the previous two reports concerning an absent FCR did not describe the existence of the FCRB, it seems to play a role in wrist flexion as an alternative to the FCR. Surgeons should have a detailed knowledge of the range of possible anomalies to complete the fixation of a distal radius fracture safely [5].

Although the FCR approach is commonly used for fixation of distal radius fractures because of its simplicity and safety, various types of anatomical variations and anomalies of the distal volar forearm structures including the median nerve, the PCB of the median nerve, and the flexor tendon have been reported. Because the FCR is designated as a favorable landmark because of its superficially palpable location, strong and thick structure, and rare anatomical variations, there is the possibility of iatrogenic complications in cases where the FCR is absent. We suggest that surgeons should have a detailed knowledge about the range of possible anomalies to complete the fixation of a distal radius fracture safely.

## Abbreviations

FCR: flexor carpi radialis
PCB: palmar cutaneous branch
PL: palmaris longus
FPL: flexor pollicis longus
PQ: pronator quadratus
FCRB: flexor carpi radialis brevis

## References

[1] Koval KJ, Harrast JJ, Anglen JO, Weinstein JN (2008). Fractures of the distal part of the radius. The evolution of practice over time. Where’s the evidence?. J Bone Joint Surg Am.

[2] Alluri RK, Hill JR, Ghiassi A (2016). Distal radius fractures: approaches, indications, and techniques. J Hand Surg Am..

[3] Conti Mica MA, Bindra R, Moran SL (2016). Anatomic considerations when performing the modified Henry approach for exposure of distal radius fractures. J Orthop..

[4] Jones C, Beredjiklian P, Matzon JL, Kim N, Lutsky K (2016). Incidence of an anomalous course of the palmar cutaneous branch of the median nerve during volar plate fixation of distal radius fractures. J Hand Surg Am..

[5] Lee YM, Song SW, Sur YJ, Ahn CY (2014). Flexor carpi radialis brevis: an unusual anomalous muscle of the wrist. Clin Orthop Surg..

[6] Bishop AT, Gabel G, Carmichael SW (1994). Flexor carpi radialis tendinitis. Part I: operative anatomy. J Bone Joint Surg Am.

[7] Rumball KM, Tonkin MA (1996). Absence of flexor carpi radialis. J Hand Surg Br..

[8] Sofos SS, Riaz M (2016). Absence of flexor carpi radialis during an elective carpometacarpal arthroplasty of the thumb: a rare anatomical variation. Case Rep Med.

[9] McCann PA, Amirfeyz R, Wakeley C, Bhatia R (2010). The volar anatomy of the distal radius—an MRI study of the FCR approach. Injury.

[10] Davis DI, Baratz M (2010). Soft tissue complications of distal radius fractures. Hand Clin.

[11] Kang L, Carter T, Wolfe SW (2006). The flexor carpi radialis brevis muscle: an anomalous flexor of the wrist and hand. A case report. J Hand Surg Am..

